# Mass Spectrometry Imaging for Mapping the Spatial Distribution of Bioactive Compounds in Medicinal Fungi: Applications, Challenges, and Future Perspectives

**DOI:** 10.3390/jof12100749

**Published:** 2026-10-07

**Authors:** Hui Fan, Suzhen Dong, Yun Zhao

**Affiliations:** 1 Shanghai Key Laboratory of Green Chemistry and Chemical Processes, School of Chemistry and Molecular Engineering, East China Normal University, Shanghai 200062, China; 2 Shanghai Engineering Research Center of Molecular Therapeutics and New Drug Development, School of Chemistry and Molecular Engineering, East China Normal University, Shanghai 200062, China; szdong@brain.ecnu.edu.cn (S.D.); yzhao@brain.ecnu.edu.cn (Y.Z.)

**Keywords:** mass spectrometry imaging (MSI), medicinal fungi, spatial distribution, bioactive compounds, sample preparation

## Abstract

Mass spectrometry imaging (MSI), featuring label-free detection, minimal sample pretreatment, and simultaneous multi-constituent analysis, overcomes the loss of spatial information inherent in conventional homogenization–extraction methods, enabling in situ visualization of bioactive constituents. This review systematically summarizes the recent advances and future perspectives of MSI in medicinal fungi, highlighting typical applications in Basidiomycota (e.g., *Lentinula edodes*, *Ganoderma* spp.) and Ascomycota (e.g., *Ophiocordyceps sinensis*, *Cordyceps cicadae*), which demonstrate the expansion of MSI from fundamental spatial metabolite localization to cultivar authentication and informing biosynthetic pathway investigations. We further analyze the critical challenges in medicinal fungal sample preparation—such as irregular morphological sizes and heterogeneous tissue structures—along with their resolution strategies. Subsequently, we outline the future directions of MSI, including ultra-high-resolution imaging, ion mobility integration, and spatial multi-omics fusion. Ultimately, MSI is propelling medicinal fungi research into a new era of “spatial metabolomics,” providing a crucial molecular spatial perspective for the precise exploitation of fungal resources, quality control, and the deep decoding of biosynthetic mechanisms.

## 1. Introduction

Medicinal fungi are an important source of natural drugs and a distinctive component of traditional Chinese medicine. Highly valued species, such as *Ganoderma lucidum*, *Ophiocordyceps sinensis*, and *Hericium erinaceus*, are rich in bioactive compounds—including triterpenoids, polysaccharides, nucleosides, and alkaloids—that mediate immunomodulatory, antitumor, antioxidant, and hepatoprotective activities [1,2]. Currently, their chemical analysis relies mainly on conventional gas or liquid chromatography coupled with mass spectrometry (e.g., GC-MS, LC-MS, UHPLC-Orbitrap MS) [2,3,4,5,6,7,8,9]. Although these techniques provide high sensitivity and broad compound coverage for precise quantification and structural identification, they require laborious solvent extraction and purification. This destructive homogenization mixes constituents from different tissue regions, erasing the original spatial distribution of metabolites and leaving traditional medicinal experience—such as the notion that the pileus of *G. lucidum* is superior to the stipe—without a scientific basis. The loss of spatial information remains a major bottleneck for precise resource development, quality control, and pharmacological investigation of medicinal fungi.

MSI offers a direct solution to this bottleneck. As a label-free in situ analytical technique, it scans tissue sections directly without extraction, simultaneously providing qualitative, semi-quantitative, and spatial information for diverse metabolites [10]. Recent advances in MSI instrumentation—such as nanoSIMS and MALDI-2—have pushed spatial resolution toward the single-cell and even subcellular level, while IMS has markedly enhanced in situ isomer discrimination [11,12,13,14,15,16,17,18]. With these capabilities, MSI has become an essential technique in modern scientific research, finding increasing application not only in natural product research, quality evaluation of traditional Chinese medicine and drug metabolism studies [10,19,20,21,22,23,24] but also in botany, food and nutrition, microbiology, biomaterials, environmental monitoring and pesticide toxicology [25,26,27,28,29,30,31,32,33,34,35,36].

Although MSI is well-established in plant and animal research, its application to medicinal fungi remains in its early stages [37]. Owing to their diverse bioactive constituents and distinctive tissue architectures, medicinal fungi such as *G. lucidum* and *O. sinensis* have gradually become a new focus for MSI-based spatial metabolomics. To the best of our knowledge, no review has yet specifically addressed MSI application in medicinal fungi. Here, we summarize the current applications of mainstream MSI techniques (e.g., MALDI-MSI, DESI-MSI, and SIMS imaging, Table 1) [10,29] in mapping the spatial distribution of bioactive compounds in medicinal fungi from both Basidiomycota (e.g., *Lentinula edodes* and *G. lucidum*) and Ascomycota (e.g., *O. sinensis* and *Cordyceps cicadae*), discuss the major challenges and corresponding resolution strategies, and outline the future perspectives. Ultimately, this work aims to provide a technical reference for the precise utilization and modernization of medicinal fungi resources.

## 2. Applications of Mass Spectrometry Imaging in Medicinal Fungi

**Figure 1:**
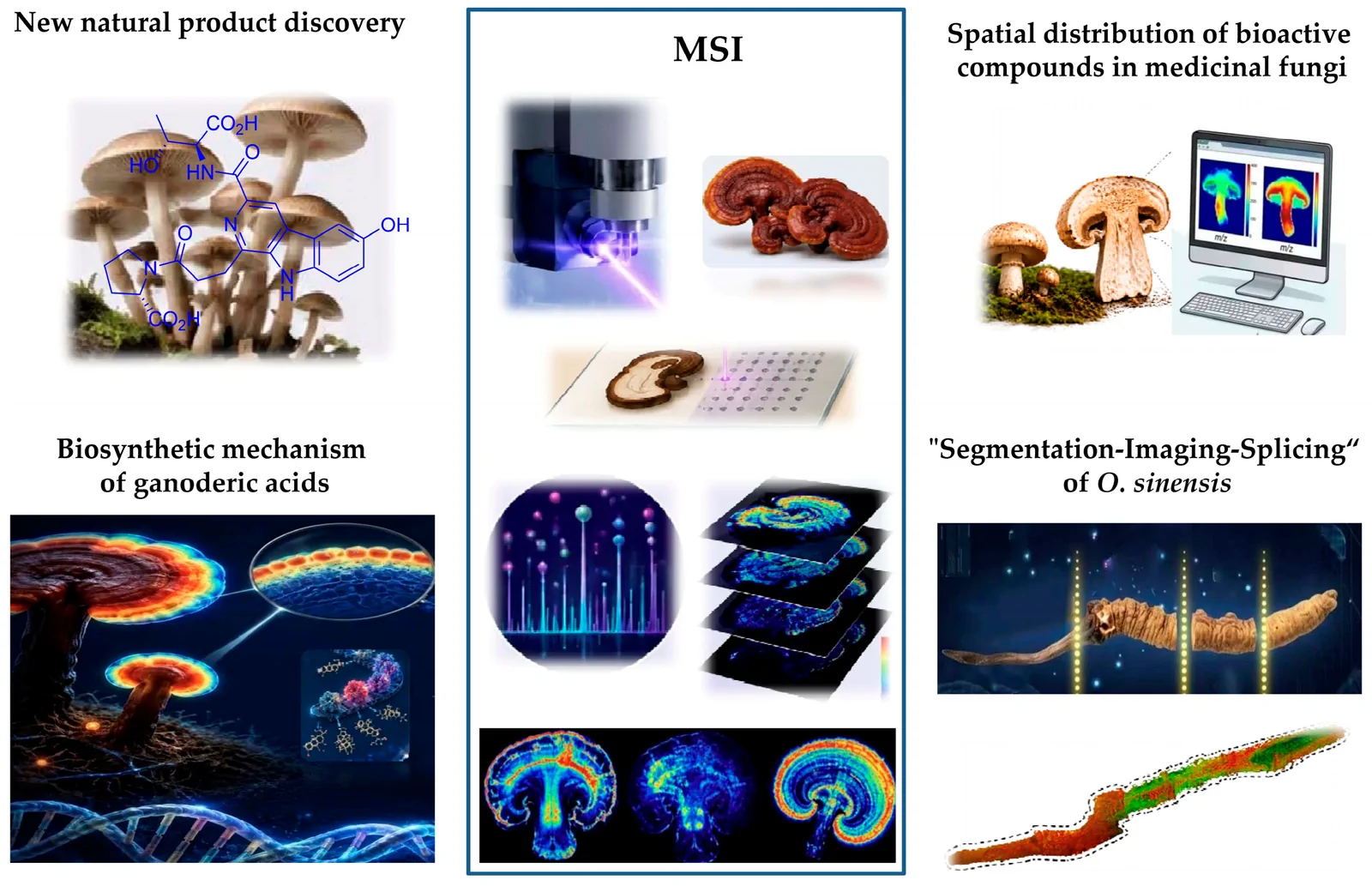
Schematic diagram of the main applications of MSI in medicinal fungi.

The medicinal fungi covered in this review mainly fall into two phyla: Basidiomycota and Ascomycota. Representative applications are summarized in Table 2 and Figure 1.

### 2.1. MSI Studies of Medicinal Fungi in Basidiomycota

Within Basidiomycota, species of Agaricales (e.g., *L. edodes* and *Agaricus bisporus*) and Polyporales (e.g., *Ganoderma lingzhi* and *Antrodia camphorata*) have successively become the subjects of MSI investigations, and their research progress is reviewed below according to taxonomic classification.

#### 2.1.1. Agaricales

(1) Edible and Medicinal Agaricales Fungi

As early as 2013, Jaeger et al. [38] combined HR-MALDI-MSI with LC-HR-ESIMS to investigate secondary metabolites in the fruiting bodies of *Mycena metata*, isolating and identifying 16 new *β*-carboline alkaloids (metacarbolines, 1a–1i and 2a–2g), and achieving the first tissue-level spatial localization of the major component (6-hydroxymetatacarboline D). MSI showed that this compound was highly enriched in the upper tissue layers of the cap, with abundance increasing from the rim to the center. This study was the first application of MALDI-MSI to the in situ screening and spatial localization of secondary metabolites in mushroom fruiting bodies, providing a new strategy for discovering novel compounds from fungi with limited biomass. Subsequently, MALDI-MSI was applied to map cyathane-type diterpenoids (striatals and erinacines) in *Cyathus striatus* and *H. erinaceus* [39]. At a spatial resolution of 15 μm and a mass accuracy below 2 ppm, the technique resolved species-specific distribution patterns: striatals A–D were uniformly distributed in the mycelial pellets of *C. striatus*, whereas erinacines in *H. erinaceus* mycelial pellets exhibited a concentration gradient decreasing from the center to the periphery, possibly reflecting growth time, nutrient diffusion, or substance migration. Notably, trace erinacines were detected for the first time in *H. erinaceus* fruiting bodies and hericenones in mycelial pellets, revising the traditional view that hericenones exist exclusively in fruiting bodies and erinacines only in mycelia.

In 2024, Vats et al. [40] used MALDI-MSI to visualize the spatial distribution of flavor-related metabolites (e.g., amino acids, organic acids, lipids) in *A. bisporus*. In fresh mushrooms, sweetness- and umami-associated amino acids (e.g., glutamic acid, aspartic acid, glutamine) were mainly enriched in the stipe and gills; after heat treatment at 140 °C, some amino acids migrated significantly toward the pileus and gills, demonstrating that thermal processing can markedly alter the spatial distribution of flavor compounds. Recently, Lu et al. [41] integrated DESI-MSI with untargeted metabolomics (UHPLC-Orbitrap MS) and multiple machine learning algorithms for the cultivar authentication and geographical traceability of three *L. edodes* cultivars (Q2, K2, S1). DESI-MSI visually mapped the spatial distribution of 65 metabolites (including amino acids, organic acids, nucleosides, and phenolics) across the pileus, gills, and stipe, revealing cultivar-specific in situ distribution differences (e.g., riboflavin mainly in the stipe and pileus center of Q2, but confined to the central cortex of the pileus in K2). Based on seven key metabolic markers (e.g., D-mannitol, L-malic acid, riboflavin, and *p*-coumaric acid) selected by complementary machine learning algorithms (RF, PLS-DA, LASSO, and RFE), a cultivar-specific “chemical–spatial fingerprint” was constructed. Notably, the classification models were validated using a dual strategy of a 70%/30% training/testing split and 5-fold stratified cross-validation, combined with permutation testing to guard against overfitting [41]. However, as this proof-of-concept study was based on only three cultivars with limited biological replicates, the translation of such machine learning models requires caution: rigorous training and validation schemes, coupled with prospective validation on sufficiently large and independent cohorts (encompassing varied growth stages, cultivation substrates, and harvest years), remain imperative before these spatial biomarkers can be routinely deployed for cultivar authentication, quality control, and precise harvesting decisions.

(2) Non-Edible Agaricales Fungi with Pharmacological or Toxicological Activities

MSI has also been applied to map pharmacological or toxicological secondary metabolites in non-edible Agaricales fungi. Hallucinogenic *Psilocybe* mushrooms are a representative example. Their principal active components, psilocybin and its analogues (e.g., psilocin, baeocystin, tryptophan, tryptamine, and aeruginascin), are highly susceptible to degradation under conventional detection conditions. Wang et al. [42] developed a CsCl-coated MALDI-FT ICR MSI method to prevent sample degradation and achieved the first in situ imaging and semi-quantitative analysis of tryptamine derivatives in these mushrooms. Psilocybin and its analogs were mainly enriched in the caps, at approximately twice the content found in the stalks.

Similarly, DESI-MS/MS imaging revealed that illudin S, a toxic constituent with antitumor activity in *Omphalotus illudens*, was highly concentrated in the stipe, up to 7.4-fold higher than in other regions [43]. In the toxic mushroom *Tricholoma kakishimeji*, MALDI-MSI was applied to visualize the spatiotemporal distribution of ustalic acid (UA) and related metabolites in its fruiting bodies [37]. UA was highly enriched on the outer surface (periphery and epidermis), with lower levels in the pileus and stipe, and its content in young fruiting bodies was significantly higher than in mature ones. This dual “spatial and developmental” dynamic pattern suggests that UA may serve as a chemical defense substance protecting young fruiting bodies from predation. Two years later, the same team developed a PESI-MS/MS method for UA detection [53], complementing MALDI-MSI and expanding its applications in fungal toxicology and food safety.

#### 2.1.2. Polyporales

In Polyporales, MSI research has focused mainly on Ganoderma species and their characteristic bioactive triterpenoids. Pioneering work by Huang et al. [44] utilized ELDI/MS to directly image hard, thick sections (1–5 mm) of *G. lucidum* and *A. camphorata*. Triterpenoids in *G. lucidum* were mainly localized at the slice surface, whereas non-volatile triterpenoids in *A. camphorata* were concentrated at both ends of the slices, and the volatile component 2,4,5-trimethoxybenzaldehyde (TMBA) was uniformly distributed across the entire surface. Although the spatial resolution (~250 μm) and imaging speed were constrained by the hardware available at that time, this work laid a crucial foundation for chemical profiling of fungal natural products. Recent advancements have enabled precise spatiotemporal profiling. DESI-MSI was applied to analyze metabolites in the pileus and stipe of *G. lingzhi* fruiting bodies across four developmental stages (squaring, opening, maturation, harvesting stage) [45]. Most ganoderic acids were significantly more abundant in the pileus than in the stipe, being predominantly enriched at the bottom edge of the mediostratum layer, and with fruiting body maturation, their levels gradually decreased, whereas fatty acids mainly accumulated in the pileus during the opening and maturation stages. These dynamic maps provide direct evidence for determining harvest time and medicinal parts.

Crucially, MSI is driving mechanistic breakthroughs. Du et al. [46] used MALDI-MSI to reveal that 24 ganoderic acids were mainly enriched in the epidermal shell of *G. lingzhi* fruiting bodies. By assembling a telomere-to-telomere (T2T) genome and constructing the first high-resolution single-cell transcriptomic atlas of the fruiting body, they reconstructed epidermal cell development. Integrated multi-omics analysis further identified and validated *GlCYP512A3*, a novel cytochrome P450 enzyme that oxidizes the intermediate GA-HLDOA to yield ganolucidic acids E and F, with its catalytic activity confirmed by in vitro enzymatic assays using engineered yeast microsomes [46]. This work advanced the understanding of the ganoderic acid biosynthetic pathway and marked a leap of MSI in medicinal fungi research from “spatial localization description” to “in-depth mechanistic elucidation.” Similarly, MALDI-MSI analysis of *G. leucocontextum* localized 18 new anti-hepatoma triterpenoids (ganoleucocontins A−R) to the epidermal shell of the pileus and stipe [47], consistent with the spatial distribution pattern reported by Du et al. [46]. Nevertheless, although this cross-species consistency suggests that the shell may serve as a key accumulation site for *Ganoderma* triterpenoids, whether it also represents an active site of biosynthesis remains to be further confirmed, as the biosynthetic role of the shell has so far been supported mainly by transcriptomic and enzymatic evidence in *G. lingzhi* [46].

Beyond profiling medicinal fungi tissues, MSI has been employed to evaluate the in vivo pharmacological mechanisms of fungal extracts. Using DESI-MSI on mouse brain tissue sections, researchers visualized that *ganoderma lucidum* polysaccharides (GLP) intervention effectively restored the disrupted cerebral metabolic homeostasis (choline, carnitine, and polyamine pathways) induced by formaldehyde exposure [54], demonstrating the potential of MSI in the pharmacodynamic evaluation of medicinal fungi.

### 2.2. MSI Studies of Medicinal Fungi in Ascomycota

The unique fungus-insect composite structure of Ascomycota species such as *O. sinensis* and *C. cicadae* complicates spatial metabolic profiling, yet MSI has recently enabled in situ visualization of their bioactive constituents.

#### 2.2.1. Spatial Distribution of Chemical Constituents in *O. sinensis*

Owing to the scarce and expensive natural resources of *O. sinensis* (formerly *C. sinensis*), elucidating the spatial distribution of its chemical components is essential for quality control and resource development. To date, three MSI studies have reported in situ imaging of its chemical constituents.

In 2022, Liu et al. [48] developed a double-layer embedding method (10% gelatin + OCT) to obtain flat, migration-free frozen sections from dried *O. sinensis* and performed TOF-SIMS imaging on both natural and artificially cultivated specimens. Over 200 chemical constituents were identified and visualized, including amino acids, nucleosides, monosaccharides, fatty acids, and glycerides. Most constituents (e.g., amino acids, monosaccharides, nucleosides, and sterols) were uniformly distributed within the caterpillar body, whereas some lipids (e.g., triacylglycerols, diacylglycerols, monoacylglycerols, free fatty acids) were mainly localized outside the host digestive tract. The same year, Xia et al. [49] proposed a “Segmentation-Imaging-Splicing” strategy for TOF-SIMS imaging to address the difficulty of sectioning and scanning whole *O. sinensis* specimens (irregular in shape and up to several centimeters in length). By sectioning and scanning the specimens in segments and computationally stitching the normalized data, they reconstructed a complete spatial map. The result showed that ergosterol was significantly more abundant in the host caterpillar than in the fruiting body, prominently enriched at the caterpillar head (the stroma junction), and exhibited a complementary spatial distribution with lipids.

More recently, MALDI-MSI combined with LC-MS was employed for in situ metabolomic and lipidomic analysis of *O. sinensis* and its host larva [50]. A total of 102 compounds were identified, revealing that various lipids (e.g., glycerophospholipids, fatty acids, sphingolipids) exhibited heterogeneous spatial distributions across the host larva, sclerotium, and stroma. The metabolites could be classified into four spatial distribution types (present only in the host larva, only in sclerotium and/or stroma, in both host larva and sclerotium, and in both host larva and stroma). During the developmental transition from host larva to *O. sinensis*, small-molecule metabolites (e.g., adenosine, hypoxanthine) increased significantly in both diversity and abundance. Rather than directly elucidating the dynamic fungal invasion mechanism or route—which would require temporal histological tracking, spatial transcriptomics, or isotope tracing—these spatial profiling data capture the terminal metabolic reprogramming and lipid turnover associated with the fungus–larva interaction. Together, these complementary MSI studies [48,49,50] collectively establish a comprehensive spatial chemomics landscape across different anatomical regions and developmental states of *O. sinensis*.

#### 2.2.2. Spatial Distribution of Metabolites in *C. cicadae*

In *C. cicadae*, another fungus-insect composite, Cao et al. [51] utilized DESI-MSI to map spatial metabolite heterogeneity across the sclerotium and coremium. A total of 62 metabolites—including nucleosides, amino acids, organic acids, and fatty acids—were annotated and characterized. Importantly, while accurate mass measurements enable putative annotation, structural validation and discrimination of isobaric/isomeric species (such as leucine/isoleucine or sugar isomers) typically rely on tandem mass spectrometry (MS/MS) fragment patterns or orthogonal LC-MS validation. The resulting MSI distribution revealed clear spatial compartmentalization: nucleosides (e.g., guanosine, adenosine), sugars and sugar alcohols, and essential amino acids (e.g., L-threonine, DL-isoleucine) were primarily enriched in the coremium; peptides (e.g., beauvericin) were localized predominantly in the sclerotium; while organic acids and fatty acids were more abundant in the core region of the sclerotium.

#### 2.2.3. MALDI-MSI Analysis of Ergot Alkaloids in *Claviceps purpurea*

*C. purpurea* is a parasitic microfungus that forms sclerotia (ergot) containing highly toxic yet pharmacologically valuable ergot alkaloids. Dopstadt et al. [52] employed MALDI-MSI to analyze sections of sclerotia formed by two *C. purpurea* strains (Ecc93 and Gal310), achieving the first in situ visualization of representative ergot alkaloids at the tissue level. In that study, the identity of the imaged alkaloids was confirmed by multiple orthogonal approaches: accurate mass and characteristic in-source fragments (within 10 ppm), collision-induced MS/MS performed both directly on tissue sections and on authentic standards, and parallel HPLC-HR-ESI-MS with retention time matching to standards. The highly polar ergometrine was distributed relatively uniformly throughout the sclerotium, whereas the weakly polar ergocristine was mainly enriched in the proximal region, displaying an increasing concentration gradient from the periphery to the center. These distribution differences may reflect both the physicochemical properties of the alkaloids and the developmental process of the sclerotium.

By pinpointing the tissue-specific distribution and spatiotemporal dynamics of key bioactive substances across Basidiomycota and Ascomycota—such as *Ganoderma* triterpenoids and ergot alkaloids—MSI provides critical spatial insights that support cultivar authentication and guide downstream biosynthetic pathway investigations and targeted pharmacological assessments, establishing a crucial foundation for precise medicinal resource exploitation.

## 3. Main Challenges and Strategies for MSI in Medicinal Fungi

Despite its great potential, the practical application of MSI in medicinal fungi is primarily hindered by sample preparation [14,55]. Ideal preparation should minimize analyte migration, enzymatic degradation, and exogenous contamination while ensuring a flat section surface, intact tissue structure, and excellent ionization efficiency [30]. Unlike lipid-rich mammalian tissues or typical plant tissues, medicinal fungi possess distinctive structural characteristics that severely complicate sectioning and metabolic preservation.

### 3.1. Challenges in MSI Sample Preparation for Medicinal Fungi

#### 3.1.1. Oversized and Irregularly Shaped Samples

For fungus-insect composites such as *O. sinensis*, the entire specimen can reach several centimeters in length, with a curved morphology and uneven thickness. This makes it difficult to obtain continuous, intact whole-body sections and far exceeds the single-field imaging view (usually at the millimeter scale) of instruments such as TOF-SIMS [49].

#### 3.1.2. High Structural Heterogeneity

Medicinal fungi fruiting bodies generally comprise tissue regions with markedly different textures, presenting a composite structure of hard and soft regions. For instance, the fruiting body of *G. lingzhi* comprises a hard, corky pileus crust, loose porous context, and tubular tissues [45]; the sclerotium of *C. purpurea* consists of a dense, hard cortex, a powdery soft outer region, and a lipid-rich inner region [52]. Such contrasting combinations render conventional cryosectioning highly prone to fracturing, folding, or delamination at tissue interfaces, severely compromising section integrity and spatial information accuracy.

#### 3.1.3. Extreme Water Content vs. Brittleness

Fresh medicinal fungi such as *A. bisporus* and *L. edodes* possess extremely high water content (approximately 90–92%). Conventional snap-freezing in liquid nitrogen induces the formation of abundant ice crystals, making the tissue brittle and prone to fracturing during sectioning. Particularly when preparing sections thinner than 35 μm, structures such as the pileus, gills, and stipe frequently break, making it exceedingly difficult to obtain intact sections [40,41]. Conversely, dried specimens are highly brittle. Attempting to rehydrate them prior to sectioning risks the delocalization and diffusion of water-soluble constituents [48].

#### 3.1.4. Matrix Interference

Conventional embedding media (e.g., OCT compound) provide necessary mechanical support for brittle samples but introduce severe organic background signals, masking low-mass endogenous metabolites [49].

### 3.2. Tailored Resolution Strategies

To overcome these biological and methodological hurdles, researchers have developed customized preparation protocols specifically for medicinal fungi.

#### 3.2.1. “Segmentation-Imaging-Splicing” Strategy

To image entire centimeter-scale *O. sinensis* specimens, Xia et al. [49] proposed a “Segmentation-Imaging-Splicing” strategy: the sample is longitudinally segmented into four millimeter-scale sections (tail of the host caterpillar, middle section of the host caterpillar, connecting section of fruiting body and host caterpillar, and end of the fruiting body). After independent TOF-SIMS imaging, the images are seamlessly spliced in their original spatial order through size and signal intensity normalization. This strategy successfully mapped the whole-body chemical panorama of oversized, irregular samples, breaking through the physical field-of-view limitations of the instrument.

#### 3.2.2. Multilayer Embedding Systems

Addressing both tissue brittleness and OCT-induced signal interference, Liu et al. [48] developed a double-layered embedding method for *O. sinensis*: rehydrated samples were first wrapped in a 10% gelatin inner layer (providing physical protection, preventing chemical migration, and shielding against matrix signal interference), followed by an outer OCT layer for robust mechanical support. This dual-layer approach effectively resolved the conflict between matrix interference and structural support.

#### 3.2.3. Optimizing Section Thickness

Unlike the ultra-thin sections (10–20 μm) standard for animal and plant tissues [25], medicinal fungal tissues often possess loose, porous hyphal structures with strong mechanical buffering capacities, allowing moderately thicker sections to preserve tissue integrity and enhance ion signals. For example, Du et al. [46] prepared sections up to 80 μm thick for *G. lingzhi* primordia and fruiting bodies, reducing fragmentation under high-energy laser bombardment and improving both signal intensity and signal-to-noise ratio. For high water content *A. bisporus*, 35 μm was optimal, balancing signal intensity and structural integrity without embedding [40]; for *L. edodes* in DESI-MSI analysis, the optimal thickness was 100 μm [41]. Overall, section thickness should be optimized by considering sample texture, target molecule abundance, and the imaging technique used.

#### 3.2.4. Tailored Pretreatment Approaches

Three tailored pretreatments have been discussed here. First, morphological restoration. For dried *O. sinensis*, controlled rehydration with MS-grade water followed by vacuum reshaping successfully restores the larva’s near-original morphology, thereby minimizing section peeling and metabolic shifting during sectioning [48]. Second, differential processing. Highly heterogeneous structures demand region-specific handling. For *C. cicadae*, Cao et al. [51] directly pressed the hydrated, soft stroma onto glass slides, whereas the hard sclerotium required slicing (2 mm) followed by freeze-drying. Third, thermal dehydration. To tackle freeze-fracturing caused by high water content, Vats et al. [40] heated whole *A. bisporus* mushrooms at 140 °C for 15 min before sectioning, evaporating excess water and reducing the section thickness from 35 μm to 17 μm, yielding fracture-free thin sections and significantly improving imaging quality.

Collectively, high-quality MSI analysis of medicinal fungi depends on a profound understanding of their structural heterogeneity and the development of customized sample preparation protocols. The “Segmentation-Imaging-Splicing” strategy, multilayer embedding systems, and optimized section thickness have overcome major challenges and markedly improved in situ data quality, enabling tissue- or even cellular-level localization of bioactive constituents. Continued innovation in embedding materials and pretreatment strategies will further unlock the potential of MSI for the precise exploitation and pharmacological research of medicinal fungi.

## 4. Conclusions and Perspectives

### 4.1. Summary of MSI Applications in Medicinal Fungi

MSI has emerged as a crucial research platform for deciphering the spatial distribution of chemical constituents in medicinal fungi, driving a transformative shift from conventional “qualitative and quantitative analysis” (knowing what is there) to “in situ spatial profiling” (knowing where it is) [14,55]. This transition enables answers to questions inaccessible to homogenization–extraction methods: where and in what patterns bioactive constituents are distributed within medicinal fungal tissues, and how such spatial information can inform investigations of the underlying mechanisms. The *Ganoderma* triterpenoids exemplify this progress. Their specific enrichment in the fruiting body shell has been observed by three MSI techniques (ELDI-MSI, DESI-MSI, MALDI-MSI) across three species (*G. lucidum*, *G. lingzhi*, *G. leucocontextum*) by four independent groups [44,45,46,47]. This cross-platform and cross-species consistency indicates that the shell serves as a key accumulation site for *Ganoderma* triterpenoids. Whether the shell also represents an active site of biosynthesis or a selective transport endpoint, however, requires further evidence: to date, the biosynthetic role of the shell has been supported mainly by the integrated T2T genomic, single-cell transcriptomic, and in vitro enzymatic evidence reported by Du et al. [46], whereas the other studies [44,45,47] provided spatial distribution data only without localized enzymatic or isotope-labeling validation. Nevertheless, these findings provide a solid empirical basis for precise harvesting, quality evaluation, and targeted utilization of *Ganoderma* medicinal fungi resources.

More notably, MSI is evolving from a “descriptive spatial distribution tool” into an “engine driving mechanistic elucidation”—a role realized when MSI is integrated with orthogonal biological tools. For example, MALDI-MSI was integrated with T2T genomics and single-cell transcriptomics, using the triterpenoid spatial pattern revealed by MSI as “spatial navigation” to identify the key CYP450 gene *GlCYP512A3* involved in ganoderic acid biosynthesis, whose catalytic function was subsequently confirmed by in vitro enzymatic assays [46]. This “spatial-first, mechanism-following” paradigm holds promise for broad applicability, although its generalizability to other medicinal fungi remains to be further explored. Furthermore, MSI has opened a new dimension for quality evaluation and species authentication: in *O. sinensis* studies, it translated the traditional medicinal wisdom that “different parts possess distinct therapeutic effects” into explicit spatio-chemical evidence [49]; in *L. edodes*, the “chemical–spatial fingerprint” demonstrated that spatial localization differences across cultivars can serve as discriminative information even when overall abundances are similar [41]. Nevertheless, rigorous validation on larger, independent, and multi-batch sample sets, together with careful prevention of machine learning overfitting, remains essential before such spatial fingerprints can be routinely deployed for quality control and cultivar authentication.

### 4.2. Future Directions and Perspectives

Despite its profound potential, the widespread application of MSI in medicinal fungi remains challenged by technical bottlenecks across spatial resolution, isomer differentiation, multi-modal integration, and massive data processing. Moving spatial fungal metabolomics forward requires not only ongoing technological innovation but also a realistic appraisal of practical trade-offs.

#### 4.2.1. Simultaneous Enhancement of Resolution and Sensitivity

Emerging high-resolution ionization platforms, such as NanoSIMS, MALDI-2, and fs-LDPI-MSM, are overcoming the trade-off between spatial resolution and sensitivity. For instance, MALDI-2 enhances ion yields by 2 to 3 orders of magnitude through secondary laser-induced post-ionization, achieving submicrometer-scale imaging [14]; the fs-LDPI-MSM system achieves 780 nm imaging resolution and attomole-level detection sensitivity while reducing full-field imaging time from hours to seconds [56]. These advances will propel metabolite localization in medicinal fungal tissues toward the single-cell and even subcellular level. However, despite their potential, ultra-high-resolution MSI faces practical limits in medicinal fungi. Sensitivity falls at micro-volume scales as shrinking pixels reduce analyte molecules and worsen signal suppression by fungal matrices. Throughput suffers: sub-micron scanning of macroscopic fungal tissues requires millions of laser shots, extending acquisition to hours or days per slide, while NanoSIMS demands laborious preparation. Molecular coverage can also be compromised, as high-energy ion beams fragment intact high-mass metabolites. Researchers must therefore balance spatial resolution against molecular coverage and throughput.

#### 4.2.2. High-Resolution Ion Mobility Spectrometry for Isomer Differentiation

Medicinal fungi produce an exceptionally rich pool of structural and stereoisomers (e.g., triterpenoid epimers in *Ganoderma* and ergot alkaloid isoforms in *Claviceps*) that cannot be differentiated by accurate mass alone [14,55,57]. Integrating high-resolution ion mobility spectrometry (IMS) provides an orthogonal separation dimension based on collision cross-section (CCS). Techniques such as DESI-cIM-MS imaging [57] and nano-DESI-TIMS-MSI [58] have successfully resolved isomeric compounds in complex biological systems and are poised to become standard approaches for accurate metabolite identification in medicinal fungi. Nevertheless, validated CCS libraries for fungal natural products remain scarce, and in silico predictions carry errors, limiting automated annotation. Moreover, ion mobility can reduce ion transmission, compromising sensitivity and throughput.

#### 4.2.3. Deep Integration of Spatial Multi-Omics and Multimodal Imaging

A single chemical profile is insufficient to decode complex fungal metabolic networks. Deep integration of MSI with spatial transcriptomics, single-cell genomics, and multimodal imaging platforms (e.g., SIMS–SEM, MALDI–FT-ICR) offers unprecedented opportunities to link biosynthetic gene expression with localized chemical outputs in medicinal fungi [14,46]. However, the different omics layers differ markedly in spatial resolution, sensitivity, molecular coverage, and sample requirements, making pixel-level or cell-level data registration a major technical hurdle. Moreover, the cost and instrumentation burden of running multiple spatial modalities on the same sample are considerable, and metabolite annotation remains the weakest link in such integrative analyses. These challenges must be addressed before deep spatial multi-omics and multimodal imaging can deliver their full promise in medicinal fungi.

#### 4.2.4. AI-Empowered Mining of Massive Datasets

A single MSI experiment often generates gigabytes (GB) to terabytes (TB) of highly heterogeneous spatial data. Artificial intelligence (AI) and machine learning are thus becoming keys to unraveling this complexity. AI technologies not only streamline upstream spectral preprocessing (e.g., denoising, baseline calibration), peak alignment, and compound annotation, but also employ spatial clustering and pattern recognition to automatically extract spatial distribution patterns and potential spatial biomarkers imperceptible to the naked eye. These AI-driven tools provide powerful support for the quality evaluation of medicinal fungi and the screening of bioactive compounds. However, training robust models requires large, well-annotated, and ideally multi-laboratory datasets, which are currently lacking for medicinal fungi; the risk of overfitting is high when model complexity exceeds sample size; deep learning approaches also demand substantial computational resources that may be prohibitive for many laboratories; and AI-assisted spectral deconvolution and automated molecular networking tools risk producing false annotations. Overcoming these challenges will enable substantial advances in the application of AI to MSI data.

Looking ahead, fueled by ultra-high-resolution hardware, ion mobility, multi-omics and multimodal fusion, and AI algorithms, MSI is propelling medicinal fungal research into a dynamic era of “spatial metabolomics”. With coordinated efforts in method development, standardization, and open data sharing, MSI will ultimately provide an irreplaceable molecular spatial perspective for the precise exploitation, quality control, and scientific modernization of medicinal fungi.

## Figures and Tables

**Table 1:** Comparison of MSI techniques commonly used in medicinal fungi.

MSI Techniques	MALDI-MSI	DESI-MSI	SIMS Imaging
Spatial resolution	5–50 μm (AP-MALDI ~1.4 μm)	50–200 μm (nano-DESI ~8–12 μm)	0.1–5 μm (NanoSIMS ~50 nm, elemental/isotope)
Operating environment	Vacuum (ambient for AP-MALDI)	Ambient	Ultra-high vacuum
Mass range (*m*/*z*)	100–100,000^+^ (depending on mass analyzer; metabolites to proteins)	50–2000 (small molecules)	<1500 (intact molecules); no such limit for elemental/isotope imaging
Sample preparation	Cryosectioning + matrix coating; relatively laborious	Minimal; matrix-free	Cryosectioning/freeze-drying; ultra-flat surface; matrix-free
Main advantages	Broad molecular coverage; soft ionization; high sensitivity; MS/MS compatible	Ambient operation; matrix-free; minimal pretreatment; near in situ; high throughput	High spatial resolution (single-cell/subcellular); isotope/elemental imaging; matrix-free
Main limitations	Low-mass matrix interference; matrix crystallization; vacuum required; complex sample preparation	Lower spatial resolution; possible analyte delocalization; moderate sensitivity; limited for nonpolar/large molecules	Extensive molecular fragmentation; narrow mass range; low throughput; strict vacuum/sample surface requirements

**Table 2:** The application of MSI in medicinal fungi.

Samples	Analytes	MSI Techniques	Section Thickness	References
*Mycena metata*	6-Hydroxymetatacarboline D	MALDI-MSI	\	[38]
*Cyathus striatus*, *Hericium erinaceus*	Cyathane-type diterpenoids	MALDI-MSI	20 μm	[39]
*Agaricus bisporus*	Flavor-related metabolites (e.g., amino acids, organic acids, lipids)	MALDI-MSI	35 μm	[40]
*Lentinula edodes*	65 Metabolites (e.g., amino acids, organic acids, nucleosides)	DESI-MSI	100 μm	[41]
*Psilocybe* mushrooms	Psilocybin and its analogues (e.g., psilocin, baeocystin, tryptophan)	MALDI-FT ICR MSI	10 μm	[42]
*Omphalotus illudens*	Illudin S	DESI-MSI	20 μm	[43]
*Tricholoma kakishimeji*	Ustalic acid	MALDI-MSI	50 μm	[37]
*Ganoderma lucidum*, *Antrodia camphorata*	Triterpenoids, 2,4,5-Trimethoxybenzaldehyde	ELDI-MSI	~2 mm	[44]
*Ganoderma lingzhi*	Ganoderic acids	DESI-MSI	10 μm	[45]
*Ganoderma lingzhi*	Ganoderic acids	MALDI-MSI	80 μm	[46]
*Ganoderma leucocontextum*	Triterpenoids	MALDI-MSI	\	[47]
*Ophiocordyceps sinensis* (formerly *Cordyceps sinensis*)	Over 200 chemical constituents (e.g., amino acids, nucleosides, fatty acids)	TOF-SIMS imaging	10 μm	[48]
*Ophiocordyceps sinensis*	Sterols, lipids	TOF-SIMS imaging	10 μm	[49]
*Ophiocordyceps sinensis*	Lipids and other small-molecule metabolites (e.g., adenosine, hypoxanthine)	MALDI-MSI	14 μm	[50]
*Cordyceps cicadae*	62 Compounds (e.g., nucleosides, amino acids, organic acids, fatty acids)	DESI-MSI	2 mm (sclerotium)	[51]
*Claviceps purpurea*	Ergometrine, ergocristine	MALDI-MSI	18 μm	[52]

## Data Availability

No new data were created or analyzed in this study. Data sharing is not applicable to this article.

## Abbreviations

The following abbreviations are used in this manuscript:

MSI	Mass spectrometry imaging
GC-MS	Gas chromatography–mass spectrometry
LC-MS	Liquid chromatography–mass spectrometry
UHPLC-Orbitrap-MS	Ultra-performance liquid chromatography–orbitrap–mass spectrometry
NanoSIMS	Nanoscale secondary ion mass spectrometry
MALDI-2	Matrix-assisted laser desorption/ionization with laser-induced post-ionization
IMS	Ion mobility spectrometry
MALDI-MSI	Matrix-assisted laser desorption/ionization–mass spectrometry imaging
DESI-MSI	Desorption electrospray ionization–mass spectrometry imaging
SIMS	Secondary ion mass spectrometry
MALDI-FT ICR MSI	Matrix-assisted laser desorption/ionization (MALDI)–Fourier transform ion cyclotron resonance (FT ICR) mass spectrometric imaging
ELDI/MS	Electrospray-assisted laser desorption/ionization mass spectrometry
TOF-SIMS	Time-of-flight secondary ion mass spectrometry
LC-HR-ESIMS	Liquid chromatography–high-resolution–electrospray ionization mass spectrometry
RF	Random forest
PLS-DA	Partial least squares–discriminant analysis
LASSO	Least absolute shrinkage and selection operator
RFE	Recursive feature elimination
CsCl	Cesium chloride
DESI-MS/MS	Desorption electrospray ionization–tandem mass spectrometry
UA	Ustalic acid
PESI-MS/MS	Probe electrospray ionization tandem mass spectrometry
TMBA	2,4,5-trimethoxybenzaldehyde
T2T	Telomere-to-telomere
GLP	*Ganoderma lucidum* polysaccharides
OCT	Optimal cutting temperature compound
fs-LDPI-MSM	Femtosecond laser desorption post-ionization–mass spectrometry microscopy
CCS	Collision cross-section
DESI-cIM-MS	Desorption electrospray ionization–cyclic ion mobility–mass spectrometry
nano-DESI-TIMS-MSI	Nanospray desorption electrospray ionization MSI coupled with high-resolution trapped ion mobility spectrometry
SEM	Scanning electron microscope
AI	Artificial intelligence
